# A cross sectional study on beliefs and roles of community pharmacy professionals in preventing and managing metabolic syndrome in an Ethiopian setting

**DOI:** 10.1371/journal.pone.0244211

**Published:** 2020-12-21

**Authors:** Sewunet Admasu Belachew, Niguse Yigzaw Muluneh, Daniel Asfaw Erku, Adeladlew Kassie Netere

**Affiliations:** 1 Department of Clinical Pharmacy, School of Pharmacy, University of Gondar-College of Medicine and Health Sciences, Gondar, Ethiopia; 2 Department of Psychiatry, University of Gondar-College of Medicine and Health Sciences, Gondar, Ethiopia; 3 Centre for Applied Health Economics, School of Medicine & Menzies Health Institute Queensland, Griffith University, Queensland, Australia; Chinese Academy of Medical Sciences and Peking Union Medical College, CHINA

## Abstract

**Introduction:**

Metabolic syndrome (MetS) is a group of cardiovascular risk factors, and its prevalence is becoming alarmingly high in Ethiopia. Studies uncovered as community pharmacy professionals (CPPs) have not yet well integrated into public health programs and priorities. In low income setting like Ethiopia, evidence regarding the roles CPPs in preventing and management of MetS is dearth.

**Objective:**

The study was aimed to assess community pharmacy professionals’(CPPs) opinions about metabolic syndrome, describe their perception level towards the effectiveness of the main interventions and explore their extent of involvement in counseling patients with the metabolic syndrome in Gondar town, Northwestern Ethiopia.

**Method:**

A descriptive, cross-sectional study was conducted among pharmacists and druggists working in community medication retail outlets (CMROs) in Gondar town, northwestern Ethiopia from April 1 to May 31, 2019. Data were collected using a self-administered pre-tested questionnaire. Descriptive statistics was used to summarize different variables, and presented in tables and figure. An independent t-test and one way ANOVA (Analysis of Variance) were used to compare mean scores. A 5% level of significance was used.

**Result:**

Out of the 75 CPPs approached, 65(40 pharmacists and 25 druggists) completed the survey giving a response rate of 86.7%. Smoking cessation practice was identified to be low. There were a statistically significant difference (t = 2.144, *P* = 0.036) in the involvement towards counseling patients between CPPs who claimed to work in pharmacy (mean = 3.96 out of 5 points Likert scale) and drug stores (mean = 3.80 out of 5 points Likert scale).

**Conclusion:**

The study concluded that the overall involvement of professionals in counseling patients, opinion about metabolic syndrome, and perception towards the effectiveness of the intervention was found to be more or less positive. However, the provision of services, such as monitoring therapy, selling equipment for home blood pressure and glucose monitoring and documenting patient care services needs to be encouraged. Given proper education and training, the current study hope that community pharmacists could be an important front-line contributors to contain this emerging epidemic in Gondar town as well as in the entire nation.

## Background

Metabolic syndrome (MetS) is a group of cardiovascular risk factors. Individuals diagnosed with this syndrome usually have the following dysmetabolic abnormalities: elevated triglycerides, low high-density lipoprotein cholesterol (HDL-C), high low-density lipoprotein (LDL-C) abnormally high fasting blood glucose, elevated blood pressure and central adiposity that is evident as an increased waist circumference [1]. As repercussion of MetS surge, all causes of death increased by about 1.5 fold and the surge was by 2-folds for cardio-vascular related mortality [2]. Consumption of calorie-dense foods, sedentary lifestyle, tobacco consumption, and use of antiretroviral medications identified as main risk factors linked with the increase of MetS [3,4].

The prevalence of non-communicable diseases (NCDs) has been noted to rise globally, and the increase was reported to be disproportionally higher in resource limited settings [5]. In the last decades, the population living with MetS have been increasing in Sub-Saharan African nations attributed to various reasons [6]. According to ATP III and IDF definitions, the occurrence of MetS, in apparently healthy working population in Ethiopia was found to be 12.5% and 17.9%, respective to the reports [7]. World Health Organization (WHO) set the key future program for the public health to form handy, multidisciplinary networks of public healthcare professionals who actively engage within communities, and provide key public health services in order to improve the life expectancy of the population [8].

Among the healthcare professionals, given community pharmacy professionals (CPPs) are one of the most accessible and well positioned health professionals in the community, they could be an essential component in an effort to prevent and manage MetS. Community medicine retail outlets (CMROs), where the CPPs work in, are found in the heart of the community convenient for population consultation or visit. This characteristic feature provides a platform for more proactive engagement in various public health priorities and programs including health-promotion and a variety of preventive services [9]. Developed nations, acknowledged CPPs as one healthcare consultants and reliable sources of health information [10]. In collaboration with clinicians, they have been strongly involved in interventional programs to manage chronic diseases such as diabetes, dyslipidemia and hypertension [11,12]. A similar study reported that pharmacist-physician collaborative practice in the care of patients with the MetS increased the proportion of patients who has get rid of the syndrome after yielding a significant improvement in blood pressure control and triglycerides levels over a 6 month period [13]. Another study completed in Lebanon stated that more than 80% of CPPs agreed that they have a key role to play in weight reduction [14].

Unlike the developed nations, CPPs role in MetS prevention and management in developing countries is largely untapped [15]. This is partly because, in low income settings, CPPs have been majorly known to involve in pharmaceutical product oriented services than engaging in patient counseling centering health behavior modification [16].

MetS is becoming a great public health challenge in Ethiopia, and there is lack of evidence about the roles of CPPs experiences, skills and beliefs in preventions and management of such risk factors. In Ethiopia, CPPs are either pharmacists or druggist/pharmacy technicians. Pharmacists are university degree level after completing comprehensive pharmacy course (5 years) while druggists/pharmacy technicians are diploma level given in colleges (3 years), and the scope of practice is narrower compared to pharmacist. Pharmacists are supposed to operate the pharmacies whereas the druggists are allowed to run the drug stores/shops.

CPPs in Ethiopia are not yet rigorously integrated as one group of health care professionals in the public health workforce and their role in providing public health services is yet to be properly recognized and endorsed by public health and governmental agencies, academicians and other healthcare professionals. Evidence also showed that CPPs are mostly involved in activities pertaining to the dispensing of medications with less participation in public health activities [17,18]. Similarly, a study conducted in the main cities of Amhara region, Ethiopia revealed that many of the community pharmacy personnel participants claimed as they were not at all involved or little involved in the counseling services regarding potential risk factors of MetS including: smoking cessation (79.3%), weight management (69.6%), hypertension screening (86.9%), dyslipidemia (88.9%), suicidal committed risks (95.1%) and performed needs assessment to recognize health risks in the community (89.8%) [19]. Nevertheless CPPs play a vital role in a variety of public health priorities in developed nations, the involvement of CPPs in chronic disease screening and lifestyle counseling were found to be very low in low income settings like Ethiopia [18,20–22]

To the best of the authors’ knowledge and search, no study found in Gondar or Ethiopia that explored the CPPs awareness and involvement in preventing syndrome, managing, monitoring patients with MetS though Gondar has many drug stores and pharmacies [23]. With this, the present study was the first in its kind aimed to assess CPPs’ opinions about MetS, describe their perception level towards the effectiveness of the main interventions and explore their extent of involvement in counseling patients with the MetS regarding prevention, identification, management and monitoring of outcomes in Gondar town, Northwestern Ethiopia.

We hope that this study will add to the existing literature gap in the area of CPPs’ role in the prevention and management of metabolic syndrome, and also believed to inform policy and practice in relation to integrating CMROs in nationwide efforts of prevention and management of MetS.

## Method

### Study setting and period

The study was conducted in Gondar town, which is located about 750 KM Northwest of the capital city Addis Ababa. According to the 2007 population and housing census report, the town has an estimated population of 206,987. Gondar town has 20 community pharmacies and 35 drug stores. The study period was from April 1 to May 31, 2019.

### Study design and population

A descriptive, cross-sectional study was employed. All CPPs who had been working in Gondar town during the study period were included.

### Sample size determination and sampling procedure

All CPPs in the 55 CMROs were our sampling frame. Through convenience sampling, all consenting CPPs who were available during the study period were sampled.

### Data collection technique and management

The data collection tool used in the study was adopted from the previously peer-reviewed studies and was prepared in **English**. The questionnaire was designed to address the objectives of the study through a robust review of existing literatures surrounding the study topic. The relevant adequate modifications was made. The final self-administered questionnaire had five sections: section one; included social-demographic items (i.e. age, gender, level of education, and income etc.) and additional information (previous training status). Section two, items related to the prevalence of MetS in Ethiopia, the risk factors of the disease, the association of metabolic syndrome with other chronic illness etc. Generally, this part assesses the professionals extent of opinion towards the items listed. Section three and four; explores CPPs” level of involvement in counseling patients with MetS regarding prevention, identification, management and monitoring of outcomes. These sections included items related to weight loss through low calorie diet, increase physical activity, advice patients on the importance of routine weight, blood pressure and blood glucose monitoring and the importance of achieving the target goals etc. The last section; deemed to assess the extent of CPPs’ perception towards the effectiveness of the main intervention for diseases prevention and management. This section was comprised of items related to weight-loss program, use dietary supplements and herbals etc.

The section of the questionnaire focusing on opinion about metabolic syndrome used a Likert scale of 1–5, where “1” represented “strongly disagree”, “2” denoted “disagree”, “3” indicated “neutral”, “4” described “agree”, and “5” stood for “strongly agree”. Similarly, the parts of the questionnaire concerning involvement in advising used a Likert scale of 1–5, where “1” stood for “very uninvolved”, “2” for “uninvolved”, “3” for “uncertain”, “4” for “involved”, and “5” for “very involved”. On the other hand, the part of the questionnaire centering perceived effectiveness used a Likert scale of 1–5, where 1” denoted “very ineffective”, “2” stood for “ineffective”, “3” for “Uncertain”, “4” represented “effective”, and “5” for “very effective”.

The three principal investigators then collected data. All of the 55 CMROs in the town were taken as the study sampling frame, and all CPPs found in the CMROs by the time of data collection were invited to part of the study. All consenting CPPs were the final study sample. The data collectors went out to all CMROs and provided the self-administered questionnaire to the CPPs in the CMROs. During the visit, each data collector explained the aim of the study, assured confidentiality of the information they provided, and then requested a consent to be part of the study. In addition, the data collectors always wait aside until the questionnaire was completed.

### Data quality control

Before the commencement of the data collection, the collectors agreed and briefly discussed on the objective of the study along with the content of the tool, the data collection methods and ethical concerns.

The questionnaire was pre-tested in 20 CMROs located in nearby towns prior to the main data collection in order to assure the inclusiveness of the tool and to confirm whether it addresses the objective of the study. Then, proper modification was employed to the format. The data obtained from pre- test were not part of the final analyses.

### Data entry and analysis

Data was edited; cleaned, coded, entered, and analyzed using Statistical Package for Social Studies (SPSS) version 22 for Windows. Descriptive statistics was used to summarize socio-demographic and other characteristics. Categorical data was expressed as frequencies and percentage while quantitative variables were described using mean with standard deviation then presented with tables and graph. An independent t-test and one way ANOVA (Analysis of Variance) were used to compare mean score differences among different groups of socio-demographic variables. For variables having two groups a t-test was employed whereas for variables having more than two groups ANOVA was utilized to determine the differences. *P*-value less than 0.05 used as cut off point for determining statistical significance.

### Ethical considerations

The ethical review committee of the School of Pharmacy, College of Medicine and Health Sciences, University of Gondar approved this study. Approval number was UoG-SoP-31/2019. Consent from CMROs was obtained; verbal and written informed consent from each participants was also gained before conducting this study. Participants’ information obtained from the questionnaires was kept confidential. The data collected was kept anonymous and recorded in such a way that involved CPPs and CMROs could not be identified. Participants were also informed that participation was voluntary and that they could withdraw from the study at any stage if they desired.

### Operational definitions

#### Pharmacy

It represents a drug shop having legal permission to hold any medicine and medical equipment. The staff who is allowed to dispense inside the pharmacy is ‘a pharmacist’ no one else is allowed to dispense according to the Ethiopian Food and Drug Administration (EFDA). Most of the pharmacies has no any separate counselling room, as a result privacy issue is one of the biggest challenge. The pharmacists need to be registered to get a license so as to open a pharmacy. The regulatory system in the area is not rigor.

#### Drug store

Unlike pharmacy, drug store is a drug shop but the medicine to be dispensed in this setting is limited that means it is not legal to hold every medications in this medicine retail outlet. For instance, it is not allowed to hold medications like psychotropic/narcotic drugs. In addition, the professional who is supposed to dispense inside the drug store is ‘a druggist ‘. All **licensing and setting standard is similar with pharmacy**.

#### Community Drug Retail Outlets (CMROs)

It is an umbrella term that denotes either or all pharmacies, drug stores, and rural drug vendors.

## Result

Among 75 CPPs approached, 65 of them completed the survey giving a response rate of 86.7%. Ten CPPs were not willing to involve in the study due to different reasons including being busy and disinterested. More than half of the respondents were druggists, 40(61.5%) while the remaining 25 (38.5%) were pharmacists. The mean age (with ± SD) of the study subjects were 28.6 ± 5.9 years. Among the participants, 35 (53.8%) were male and found to be older than females with a mean age (with ± SD) of, 31.3 ± 35 years. Majority of respondents had a work experience of >5 years, 31(47.7%) followed by 1–5 years, 27 (41.5%).

Out of 65 study subjects, only 16 (24.6%) of them took training on prevention and management of MetS. However, majority of the CPPs, 61(93.8%) claimed as they have heard about the concept of MetS. The details are further illustrated at Table 1.

**Table 1:** Socio-demographic characteristics of the respondents, Gondar, 2019, N = 65.

Variables		Frequency (%)
Sex	Male	35(53.8)
	Female	30(46.2)
Qualification	Druggist	40(61.5)
	Pharmacist	25(38.5)
Work experience (year)	<1	7(10.8)
	1–5	27(41.5)
	>5	31(47.7)
Salary per month (birr)	≤ 1499	3(4.6)
	1500–3000	27(41.5)
	>3000	22(33.8)
Work sector	Owner	13(20)
	Drug store	25(38.5)
	Pharmacy	40(61.5)
Do you know what metabolic syndrome is?	Yes	61(93.8)
	No	4(6.2)
Had training on prevention and management of metabolic syndrome	Yes	16(24.6)
	No	49(75.4)

The respondents’ opinions about the MetS as a health problem were explored after providing them with information about its definition, required components and main complications.

Majority of respondents ‘‘agreed” and ‘‘strongly agreed” as metabolic syndrome is common and showed a growing prevalence in Ethiopia, 63.1% and 23.1%, respectively. The overall mean score was determined to be 4 out of the 5 point Likert scale. With a high mean score (4.2), most of the study subjects agreed and strongly agreed (89.2%) that MetS relates largely to obesity and sedentary lifestyles. A high percentage of participants disagreed (41.5%) and remained neutral (36.9%) to statement that mentions that the public awareness towards the link between MetS and the high risk for cardiovascular diseases and type 2 diabetes is poor. This statement had the lowest mean score (2.7).

Only two respondents (3.1%) strongly disagreed with following statement “the link between metabolic syndrome and the high risk for cardiovascular diseases or other non-communicable diseases is well understood by the public in Ethiopia”. The details are described in the Table 2.

**Table 2:** Percentage distribution of community pharmacy professionals’ opinions about the metabolic syndrome, Gondar 2019, N = 65.

Items	Strongly disagree, N (%)	Disagree, N (%)	Neutral, N (%)	Agree, N (%)	Strongly agree, N (%)	Mean
Metabolic syndrome is communal and has a growing prevalence in Ethiopia	0(0)	3(4.6)	6(9.2)	41(63.1)	15(23.1)	4
Metabolic syndrome is highly associated with obesity and sedentary lifestyles	0(0)	1(1.5)	6(9.2)	37(56.9)	21(32.3)	4.2
The link between metabolic syndrome and the high risk for cardiovascular diseases or other Non-communicable disease(NCDs) is well understood by the public in Gondar	2(3.1)	27(41.5)	24(36.9)	12(18.5)	0(0)	2.7
High emphasis must be given to modifications of lifestyles of the general public in Gondar	0(0)	0(0)	9(13.8)	32(49.2)	24(36.9)	4.2
Individual patients with metabolic syndrome need to be identified early so that their multiple risk factors can be reduced	0(0)	0(0)	5(7.7)	35(53.8)	25(38.5)	4.3

Table 3 shows the extent of respondents’ involvement in advising patients regarding seven aspects of lifestyle modifications to circumvent the syndrome.

**Table 3:** Percentage distribution of community pharmacy professionals ‘involvement in advising patients with the metabolic syndrome regarding lifestyle modifications, Gondar 2019, N = 65.

Items	Very uninvolved, N (%)	Uninvolved, N (%)	Uncertain, N (%)	Involved, N (%)	Very involved, N (%)	Mean
Weight loss through low calorie diet	1(1.5)	3(4.6)	7(10.8)	39(60)	15(23.1)	3.9
Increase physical activity	0(0)	1(1.5)	7(10.8)	39(60)	18(27.7)	4.1
Salt restriction for hypertension patients	0(0)	0(0)	8(12.3)	29(44.6)	28(43.1	4.3
Cholesterol-lowering diet (composed of reduced cholesterol/saturated fat intake)	0(0)	2(3.1)	10(15.4)	32(49.2)	21(32.3)	4.1
Increase consumption of plant vegetables	0(0)	3(4.6)	18(27.7)	33(50.8)	11(16.9)	3.8
Increase consumption of soluble fiber	0(0)	1(1.5)	22(33.8)	35(53.8)	7(10.8)	3.7
Smoking cessation	0(0)	8(12.3)	32(49.2)	21(32.3)	4(6.2)	3.3

The percentages of respondents who claimed to be involved or very involved in counseling patients regarding weight loss through low calorie diet were, 60% and 23.1%, respectively. The majority of study subjects either involved or very involved in advising patients to increase physical activity (87.7%), restrict salt use (87.7%), and utilize cholesterol lowering diet: composed of reduced cholesterol/saturated fat intake (81.5%), increase consumption of plant (67.7%), and increase consumption of soluble fiber (64.6%). Regarding smoking cessation, almost half of the study participants (49.2%) claimed as they were not certain enough even whether they were involved or not. Apart from this, only a single respondent stated as ‘very uninvolved’ towards weight loss through low calorie diet with no other respondents claiming as ‘very uninvolved’ for any advises listed.

Moreover, the mean score for each advises listed has been determined. The lowest mean score was for smoking cessation (3.3) followed by advice to increase consumption of soluble fibers (3.7). Whereas, the highest mean score was for salt restriction (4.3) advices followed by advices to increase physical activity and cholesterol-lowering diet, each got (4.1).

The extent of respondents’ involvement in advising patients regarding the prevention and management of the metabolic syndrome were elaborately demonstrated.

The majority of respondents indicated that they were involved or very involved in counseling patients about the importance of routine weight, blood pressure and glucose monitoring as well as the necessity of achieving the target goals (90.8%). Also, involved or very involved in selling equipment for home blood pressure and glucose monitoring (58.5%), non-prescription treatments and self-care for the components of the MetS (90%), adherence with treatment (86.2), monitoring patients’ response to the treatment (53.8%), referring patients to physicians if required (90.8%). In regards to retaining records of patient care services in CMRO, more than half of the CPPs (53.8%) revealed as they were not sure enough whether they were involved or not.

Furthermore, the mean score for each advises were estimated. The lowest mean score went for keeping records of patient care services in the CMRO (3.3) followed by selling equipment for home blood pressure and glucose monitoring (3.5), and monitoring patients’ response to the treatment (3.6). Whereas, the highest mean score was for advising patients on the importance of routine weight, blood pressure and glucose monitoring and the prominence of achieving the target goals, both had a mean value of 4.3. The detailed information are illustrated in Table 4.

**Table 4:** Percentage distribution of pharmacy professionals’ involvement in providing services to patients for the prevention and management of the metabolic syndrome, Gondar, 2019, N = 65.

Items	Very uninvolved, N (%)	Uninvolved, N (%)	Uncertain, N (%)	Involved, N (%)	Very involved, N (%)	Mean
Counseling clients on the advantage of routine weight, blood pressure and blood glucose monitoring and the importance of achieving the target goals	0(0)	0(0)	6(9.2)	31(47.7)	28(43.1)	4.3
Selling home based blood pressure and blood glucose monitoring equipment.	2(3.1)	8(12.3)	17(26.2)	26(40)	12(18.5)	3.5
Advise patients about non-prescription treatments and self-care for the components of the metabolic syndrome	1(1.5)	3(4.6)	9(13.8)	45(69.2)	7(20.8)	3.8
Encourage patient adherence with treatment	0(0)	1(1.5)	8(12.3)	30(46.2)	26(40)	4.2
Monitor patients’ response to the treatment	0(0)	2(3.1)	28(43.1)	29(44.6)	6(9.2)	3.6
Keeping records of patient care services in the pharmacy	0(0)	6(9.2)	35(53.8)	20(30.8)	4(6.2)	3.3
Refer patients to physicians or healthcare clinics if required	0(0)	0(0)	6(9.2)	33(50.8)	26(40)	4.3

**Fig 1:**
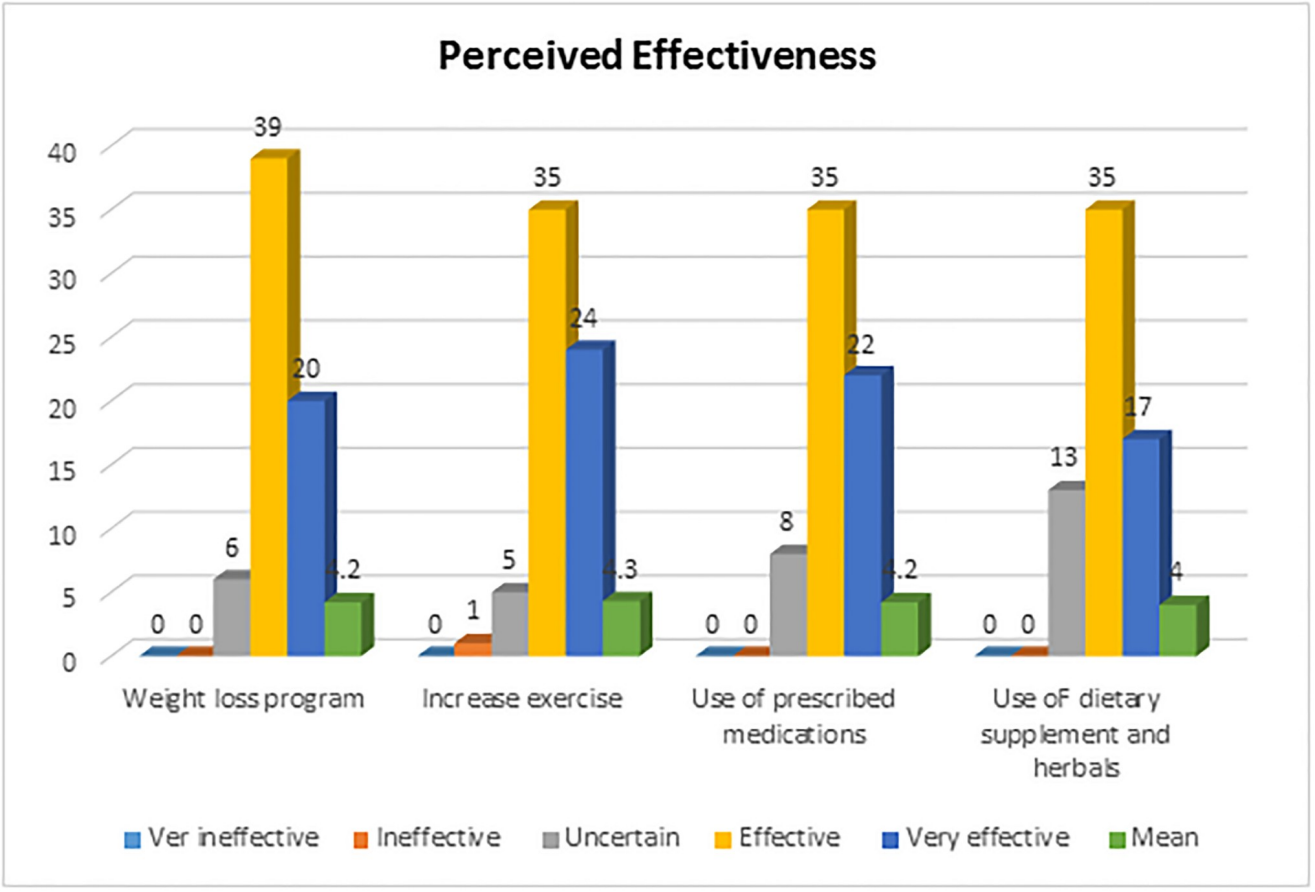
Percentage distribution of pharmacy professionals’ perceived effectiveness of some of the interventions in patients with the metabolic syndrome, Gondar, 2019, N = 65.

Fig 1 shows the pharmacists’ perceptions of the effectiveness of four main interventions used for the management of the syndrome.

Majority of the study participants ranked the listed core interventions as being ‘effective’ or ‘very effective’ (i.e. weight-loss program 59 (90.8%) increase exercise 59 (90.8%) and use of prescribed medications for the treatment of the individual components of metabolic syndrome 57(87.6%)). More than three quarter of the respondents perceived the use dietary supplements and herbals as being very ‘effective or very effective’. None of the professionals perceived all the mentioned intervention as being ‘very ineffective’. In addition, the mean scores for each intervention were found to be similar, each 4.2 out of the 5 point Likert scale with the exception one intervention that was use of dietary supplements and herbals, had a mean score of 4.

An independent sample t-test was employed to explore the mean difference among respondents in regards to overall opinion towards MetS and their involvement in counseling patients with metabolic syndrome regarding life style modifications and prevention as well as management. In addition, CPPs mean difference towards perceived effectiveness of the main intervention was also analyzed.

The overall mean involvement of CPPs in counseling patients, opinion about metabolic syndrome and perception towards the effectiveness of the main interventions was 3.90, 3.89, and 4.18 on a 5 point Likert scale, respectively. Though it was not statistically significant, the mean response of respondents towards their involvement in counseling patients (*P* = 0.197), opinion towards metabolic syndrome (*P* = 0.207) and perception towards the effectiveness of the interventions (*P* = 0.138) were found to be higher among pharmacists (3.96, 3.97, 4.29 out of the 5 points Likert scale, respectively) compared to druggists (3.86, 3.85, 4.12 out the 5 points Likert scale, respectively). Nevertheless it was not statistically significant, the mean response of respondents regarding their opinion towards metabolic syndrome (*P* = 0.785), perception towards the effectiveness of the main interventions (*P* = 0.766) were identified to be higher among professionals who claimed to have heard about MetS (3.90, 4.19 out the 5 points Likert scale, respectively) than that of their counter parts (3.75, 4.12 out the 5 points Likert scale, respectively). The mean response of respondents regarding their opinion towards MetS (*P* = 0.870), the perception towards the effectiveness of the main intervention (*P* = 0.992) were found to be higher among the study participants who took training on prevention and management of MetS (3.91, 4.21 out the 5 points Likert scale, respectively) compared to those who did not (3.86.4.16 out the 5 points Likert scale, respectively).

There were a statistically significant difference (t = 2.144, *P* = 0.036) in the involvement towards counseling patients between CPPs who claimed to work in pharmacy (mean = 3.96 out of the 5 points Likert scale) and drug stores (mean = 3.80 out of the 5 points Likert scale). The details are presented in Table 5.

**Table 5:** Statistical test (independent sample t-test) of differences among categories of respondents in overall involvement, opinion and perceived effectiveness towards metabolic syndrome management, Gondar, 2019, N = 65.

Variables	Overall involvement towards advise about lifestyle modification, prevention and management			Overall opinion towards metabolic syndrome			Overall Perceived effectiveness		
	Mean(SD)	t	*P*	Mean(SD)	t	*P*	Mean(SD)	t	*P*
Gender:									
Male	3.89(0.34)	0.208	0.836	3.85(0.38)	1.05	0.296	4.22(0.44)	0.65	0.514
Female	3.91(0.26)			3.95(0.38)			4.15(0.42)		
Qualification:									
Druggist	3.86(0.29)	1.304	0.197	3.85(0.38)	1.27	0.207	4.12(0.42)	1.501	0.138
Pharmacist	3.96(0.31)			3.97(0.39)			4.29(0.44)		
Sector:									
Drug store	3.80(0.29)	2.144	0.036*	3.82(0.43)	1.24	0.225	4.19(0.39)	0.022	0.982
Pharmacy	3.96(0.30)			3.94(0.35)			4.18(0.46)		
Know about Metabolic syndrome									
Yes	3.88(0.30)	1.633	0.107	3.90(0.37)	0.785	0.435	4.19(0.44)	0.299	0.766
No	4.14(0.35)			3.75(0.57)			4.12(0.32)		
Previous training:									
Yes	3.88(0.35)	0.289	0.774	3.91(0.44)	0.165	0.870	4.21(0.44)	0.010	0.992
No	3.90(0.30)			3.86(0.37)			4.16(0.43)		

**Note**:

*P-value less than 0.05.

A One-way ANOVA was employed to explore the mean difference among respondents in regards to overall opinion towards MetS and their involvement in counseling patients with MetS regarding life style modifications and prevention as well as management. Moreover, CPPs mean difference towards perceived effectiveness of the main intervention was also explored.

Nevertheless, it was found not to be statistically significant (*P* = 0.815), a mean difference in the involvement towards counseling patients was noted among the different groups of respondents based on salary. With this, the highest mean (3.97) was recorded for respondents who owned the medication retail outlet followed by professionals who claimed to have a salary of ≤1499 ETB (3.90), whereas the lowest mean was observed for study subjects who indicated to have a salary of ≥ 3000 ETB. The mean perception towards the effectiveness of the mentioned interventions was found to be highest (4.36 out of the 5 point Likert scale) among respondents who have had a salary of ≥ 3000 ETB while the lowest mean (3.91out of the 5 point Likert scale) was noticed among participants who had a salary of ≤1499 ETB; However, it was not statistically significant (*P* = 0.112). Whiles the highest mean (3.95 out of the 5-point Likert scale) response regarding the opinion towards MetS was seen among CPPs who owned the medication retail outlet, the lowest (3.66 out of the 5-point Likert scale) was for respondents with a salary of ≤1499 ETB.

Though it was found not be statistically significant (*P* = 0.374), a mean response difference in the perception towards main interventions was noticed among the respondents who had different work experience in terms of duration. With this, the highest mean (4.25 out of the 5 point Likert scale) was noted for respondents who had > 5years of work experience while lowest (4.0 out of the 5 point Likert scale) was for participants who had < 1 year of work experience. The details are depicted in Table 6.

**Table 6:** Statistical test (one-way ANOVA) of differences among categories of respondents in overall involvement, opinion and perceived effectiveness towards metabolic syndrome management, Gondar, 2019, N = 65.

Variables	Overall advice towards Lifestyle modification, prevention and management			Overall opinion towards metabolic syndrome			Overall Perceived effectiveness		
	Mean(SD)	F	P	Mean(SD)	F	P	Mean(SD)	F	P
Salary(birr):									
≤1499	3.90(0.29)	0.315	0.815	3.66(0.23)	0.487	0.693	3.91(0.38)	2.084	0.112
1500–3000	3.89(0.32)			3.91(0.31)			4.11(0.41)		
≥3000	3.86(0.31)			3.87(0.47)			4.36(0.40)		
Owner	3.97(0.28)			3.95(0.40)			4.11(0.47)		
Experience(year):									
<1	4.0(0.25)	2.775	0.070	3.91(0.34)	0.016	0.984	4.0(0.50)	1.00	0.374
1–5	3.80(0.28)			3.88(0.31)			4.16(0.41)		
>5	3.96(0.31)			3.90(0.46			4.25(0.44)		

## Discussion

The varying impact of community pharmacy professionals (CPPs) from their traditional core dispensing responsibilities to a greater contribution to population health is being recognized all over the world [21]. To the best of authors’ knowledge and search, this is the pioneer survey to explore the awareness and role of CPPs in preventing and managing metabolic syndrome in Gondar town and northwestern Ethiopia at large. With this, the current study was aimed to reveal the opinions of CPPs about metabolic syndrome, their extent of involvement in counseling patients with metabolic syndrome regarding prevention, identification, management and monitoring outcomes, and determine the perception level towards the effectiveness of the main interventions.

In the current study, the overall mean involvement of professionals in counseling patients, opinion about metabolic syndrome, and perception towards the effectiveness of the main interventions was 3.90, 3.89, and 4.18 on a 5- point Likert scale, respectively. All in all this this shows that; the professionals almost agreed with the basic points listed regarding metabolic syndrome, also revealed as they were almost being involved in counseling patients with metabolic syndrome regarding prevention and management, and they all perceived the main intervention to prevent and manage metabolic syndrome as beyond effective. In this study, majority of the professionals, 61(93.8%) claimed as they have heard about metabolic syndrome. This might partly be justified as the pharmacy education system have had a paradigm shift from product oriented to patient oriented approaches which in turn lets new graduate professionals working in each medication retail outlets to be informed with the different clinical cases along with their patient approach and management. Out of 65 study subjects, merely 16 (24.6%) of them got training on prevention and management of metabolic syndrome. This might be attributed to the low initiative taken by the zonal health office in training as well as updating CPPs regularly. In addition, the link of the medication retail outlets with the nearby higher education institution is minimal that experts has not been invited to train.

The current study highlighted that the majority of respondents ‘‘agreed” and ‘‘strongly agreed” as metabolic syndrome is common and got a growing prevalence, 63.1% and 23.1%, respectively. In comparable with the current study findings a study conducted in united states indicated that 75% and 61% of pharmacists believed strongly that metabolic disease such as chronic heart disease and high blood cholesterol levels, respectively, are significant health problems facing Americans [24]. Similarly, a study done in Kuwait revealed that 94.5% of community pharmacy professionals agreed and strongly agreed as metabolic syndrome is common and outgrowing in the country [25]. In addition, a study done by Via-Sosa MA et al noted as pharmacists believed that premorbid metabolic syndrome is alarmingly increasing [26]. On the other hand, most of the study subjects agreed and strongly agreed (89.2%) to state that metabolic syndrome relates largely to obesity and sedentary lifestyles. This finding concurs with the result of other study which reported that CPPs believed metabolic syndrome as a common health problem in the country that is largely related to obesity and sedentary lifestyles of the public [25]. Another study completed from Lebanon also noted that more than 80% of CPPs agreed as they have a pivotal role in weight reduction efforts [14]. A high percentage of participants disagreed (41.5%) and remained neutral (36.9%) to statement that mentions as the public awareness towards the link between metabolic syndrome and the high risk for cardiovascular diseases and type 2 diabetes is poor. This might be due to the fact that most of the clients who visit the retail outlets are uneducated, have no clue, or know little about health related issues as the places lacked public awareness campaign events. Furthermore, the professionals also believed that early identification of untreated patients is needed for risk reduction. The role of pharmacists in early identification of high-risk patients and their referral to physicians has been reported by several studies [20,27,28].

Indeed, the percentages of respondents, who claimed to be either involved or very involved in counseling patients on weight loss through low calorie diet were, 60% and 23.1%, respectively. It has been also reported in another studies that community pharmacists are well positioned to provide weight management advice to the public [19,25,29]. Moreover, the majority of study subjects involved or very involved in advising patients to increase physical activity (87.7%), restrict salt (87.7%), and utilize cholesterol lowering diet: composed of reduced cholesterol/saturated fat intake (81.5%), increase consumption of plant (67.7%) and increase consumption of soluble fiber (64.6%). These findings are inconsistent with a study done in other places, which reported as community pharmacy professionals have been the most accessible outlets to offer counseling about exercise, salt restriction, cholesterol reduction strategies etc. [25,30]. Regarding smoking cessation, almost half of the study participants (49.2%) claimed, as they were not certain enough even whether they were involved or not. In contrast, a study done in other countries publicized as community pharmacists are uniquely placed for providing public with smoking cessation service and revealed as they frequently counsel patients on smoking cessation [25,31]. This might be attributed to the high smoking rate in developed countries that the issue of tobacco use remained to be the focus of counseling in those places unlike the case of Ethiopia. In the present study, the majority of respondents indicated that they were ‘involved or very involved’ in counseling patients on the importance of routine weight, blood pressure and blood glucose monitoring and the importance of achieving the target goals (90.8%). Similarly, several studies observed that community pharmacy professionals proactively engage in advising patients regarding benefit of weight, blood pressure and glucose measurement [25,32,33].

In addition, the majority of pharmacists believed that their professional responsibilities include advising patients about nonprescription therapies and encouraging patients’ adherence with prescribed therapies. These findings are comparable with the results of other studies conducted in USA and Kuwait [24,25]. In the current study, more than half the professionals mentioned as they monitor patients’ response to the treatment. Comparably, a study done in other place indicated that three quarters of the respondents claimed to monitor patients’ response to treatment [25]. More than half of the professionals (53.8%) revealed, as they were not sure enough whether they were involved or not. This indicated that the introduction of patient care documentation systems into the community pharmacies in Gondar is needed to improve the quality of services and possibly in the future, to be used as basis for pharmacists’ remuneration.

Basically, majority of the study participants in the current study ranked weight-loss program 59 (90.8%), increasing exercise 59 (90.8%) and use of prescribed medications as being effective or very effective interventions. This is in consistent with a study done in other countries, which uncovered that majority of the professionals in community pharmacies perceived weight reduction initiatives (92.3%), increasing physical activity (94.1%) and use of prescribed medications (95%) as being effective or very effective [25]. In line with this, more than three quarter of the respondents perceived the use dietary supplements and herbals as being very effective or very effective. However, several studies contradicts the current study finding that pharmacists perceived the use of dietary supplements and herbals as being relatively less effective compared to lifestyle interventions for management of coronary heart disease and obesity [24,34]. In general, the current study highlighted the need to design and implement effective educational programs to capacitate community pharmacists to provide efficient pharmaceutical care services to patients with the metabolic syndrome.

In parallel, the current study also managed to analyze and describe the differences in overall mean score among different socio demographic characteristics. According to the independent sample t-test and one-way ANOVA result: Except one variable, all the rest variables considered for the inferential analysis were found to be insignificant. With this, there was a statistically significant difference (t = 2.144, *P* = 0.036) in the involvement towards counseling patients between professionals who claimed to work in pharmacy (mean = 3.96 out of the 5 points Likert scale) and drug stores (mean = 3.80 out of the 5 points Likert scale). This might be partially justified as it because of the difference in the setting and quality of professionals on duty. More or less the set up in pharmacy is conducive for counseling and the experts working in are usually pharmacists unlike drug stores.

### Study strengths and limitations

This study highlighted an area of community pharmacy practice where there is lack of literature in Ethiopia. In addition, as a strength the study had high response rate and used a structured questionnaire for data collection that was easily understandable by the respondents. Yet, the survey has some limitations that should be noted while interpreting the results. As far the study was a cross-sectional survey conducted in Gondar town, caution should be exercised when generalizing to other cities and regions in Ethiopia. The information given by respondents may be influenced by what is perceived to be the right answer to give. Therefore, the extent of truthful answers or verifying respondents’ claims is not possible in this type of study. In addition, pharmacists’ responses to some questions were dependent on their ability to recall experiences with patients. Moreover, our direct visit of community pharmacy professionals at their work place to provide them the questionnaire could affect the responses as it may be subjected to respondent bias, which could have been reduced had our study been simulated patient approach. Even with the above limitations, this study fills an important gap in the literature and provides useful information about the role of community pharmacists in the prevention and management of the metabolic syndrome.

## Conclusion

The overall involvement of professionals in counseling patients, opinion about metabolic syndrome, perception towards the effectiveness of the intervention was found to be more or less positive. CPPs recognized that MetS is an important health problem in Ethiopia and stated as it is surging through time. Many CPPs indicated that they are able to provide useful advises to patients about the syndrome, but the provision of services, such as monitoring therapy, consumption of soluble fiber, selling equipment for home blood pressure and glucose monitoring, and documenting patient care services needs to be further improved. Most importantly, CPPs involvement in smoking cession practice was found to be low and therefore, needs lots of efforts to be on place so as to improve CPPs’ engagement in tobacco use reduction activities.

## Implication of the findings

Bearing in mind the heightened importance of the integration of CMROs into upcoming patient care and to enhance the contribution of CPPs, interventions by ministry of health, local health policy makers, collaborator and stakeholders should put efforts to better improve the overall syndrome awareness and involvement of CPPs through establishing and providing regular tailored educational programs and refreshment trainings. In this regard, the zonal health office should work closely with the medication retail outlets to establish a strong and sustainable community pharmacy based smoking cessation service, as one strategy to tackle MetS. Furthermore, using the current study finding as an input, upcoming researchers could aim nationwide researches employing broader multi-center mixed methods such as self-reported cross-sectional, and simulated client based studies. In addition, qualitative studies would also be needed to identify barriers and facilitators to implementations of pharmacy practice in relation to MetS containment to ultimately inform policy and practice.

## Supporting information

S1 File SPSS data set. (SAV)

## Abbreviations

ANOVA: Analysis of Variance
ATP III: Adult Treatment Panel III
CMROs: Community medication retail outlets
CPPS: Community Pharmacy professionals
EPA: Ethiopian Pharmaceutical Association
ETB: Ethiopian Birr
HDL-C: high-density lipoprotein cholesterol
IDF: International Diabetic Federation
LDL-C: low-density lipoprotein-C
MetS: Metabolic syndrome
NCDs: non-communicable diseases
SPSS: Statistical Package for Social Studies
T2DM: Type 2 diabetes mellitus
